# The effect of vitamin A deficiency on the initiation and postinitiation phases of benzo(a)pyrene-induced lung tumourigenesis in rats.

**DOI:** 10.1038/bjc.1985.279

**Published:** 1985-12

**Authors:** S. C. Dogra, K. L. Khanduja, M. P. Gupta

## Abstract

**Images:**


					
Br. J. Cancer 1985), 52, 931-935

The effect of vitamin A deficiency on the initiation and
postinitiation phases of benzo(a)pyrene-induced lung
tumourigenesis in rats

S.C. Dogra, K.L. Khanduja & M.P. Gupta

Department of Biophysics, Postgraduate Institute of Medical Education and Research, Chandigarh-160 012,
India.

Summary The present investigation shows the effect of vitamin A deficiency on the initiation and
postinitiation phases of benzo(a)pyrene-induced lung carcinogenesis in male Wistar rats. Lung tumours were
induced by giving three intratracheal instillations, one week apart, of 10mg benzo(a)pyrene per instillation.
Maximum tumour incidence (100%) and tumour burden per rat was found in rats which were kept on
vitamin A deficient diet for 4 weeks prior to the first administration and 8 weeks after the last administration
of benzo(a)pyrene. Rats in which vitamin A deficiency was terminated after the last administration of the
carcinogen had 83% tumour incidence, whereas corresponding control pairfed animals had 39% incidence of
tumours. These data represent the values obtained 32 weeks after the last administered dose of the carcinogen
and indicate the role of vitamin A, both in the initiation as well as in the postinitiation phases of lung
carcinogenesis.

Today vitamin A is well known for its importance
in general growth and differentiation of epithelial
tissues and its deficiency has been shown to lead to
metaplastic changes in the epithelia of the res-
piratory, urogenital and gastrointestinal tracts
(Harris et al., 1972; Wolback, 1954). These meta-
plastic changes appeared by light microscopy to be
morphologically similar to the changes found in
certain precancerous lesions caused by carcinogen
administration (Harris et al., 1972; Hayes, 1971).
This similarity between histological features of
epithelial tissues of vitamin A deficient animals and
certain precancerous lesions was the starting point
for further investigations in this area. Several
investigators have confirmed and extended the early
observations and have shown a prophylactic effect
of vitamin A on the development of carcinogen-
induced epithelial tumours (Bollag & Matter, 1981;
Lotan, 1980; Nettesheim & Williams, 1976).
However, supplementation with retinoids as a
prophylactic agent has resulted in variable effects
and these are being related to the problem of
distribution and toxicity of vitamin A (Sporn,
1976).

Nettesheim et al. (1976) investigated the influence
of retinyl acetate supplementation at high dose on
the initiation and postinitiation phase of 3-methyl-
cholanthrene induced preneoplastic lung nodules in
rats. In this study the incidence of metaplastic lung
nodules was found to be 3% in the combined high

Correspondence: K.L. Khanduja.

Received 23 March 1985; and in revised form 17 August
1985.

retinyl acetate dose groups as compared with 42%
in the low retinyl acetate dose group, and it was
concluded that retinyl acetate treatment had a
significant inhibitory effect on the postinitiation
phase of preneoplastic lung nodules in rats.
However, the effect of vitamin A deficiency on the
initiation and postinitiation phases of carcino-
genesis has not been evaluated. Therefore, the
primary objective of the present investigation was
to study and compare the effect of benzo(a)pyrene,
a potent environmental lung carcinogen, on tumour
incidence in vitamin A deficient rats, both at the
initiation and postinitiation phases of pulmonary
carcinogenesis.

Materials and methods

Male weanling rats (45-60 g) of our Institute's
colony (Wistar derived) were used. They were
maintained for 4 weeks on vitamin A deficient
casein based diet composed of vitamin-free casein
(20%), corn starch (39.8%), dextrose (30%), refined
peanut oil (5%), salt mix (4%), choline chloride
(0.2%), and vitamin A-free vitamin mix (1.0%). An
identical diet supplemented with retinyl acetate
(20,000 IU kg- 1) was given to control animals.
Animals had free access to water and food during
the first 4 weeks, thereafter during the next 2 weeks
the amount of diet supplied to the control rats was
reduced in proportion to that consumed by the
deficient group.

Equal amounts of benzo(a)pyrene (BP) and
Fe203 were mixed in a mortar and ground together

(j The Macmillan Press Ltd., 1985

932     S.C. DOGRA et al.

for 2 - 4 h, yielding a fine, homogeneously

distributed mixture of BP and Fe2O3 (1:1) by

weight. This mixture was prepared freshly every
week before instillation and was then placed in a
25 ml flat-bottomed conical flask to which
0.15 M NaCl (sterile) was added to make the final
concentration of the mixture 50 mg ml - 1. After
maintaining rats on their respective diets for 4
weeks, a total of three intratracheal instillations
were given to each rat with a gap of one week
between each administration. Each instillation
consisted of 20mg mixture of BP-Fe203/0.4 ml/rat.
Animals were instilled under light ether anaesthesia.
One day after the third instillation, rats from the
vitamin A deficient group were randomly divided
into two groups i.e. groups 2 and 3, to receive diet
either containing 20,000 IU kg- 1 or 1000 IU kg- 1 of
retinyl acetate respectively. Rats from group 3 were
maintained   on   vitamin  A    deficient  diet
(1000IUkg-1) for a further 8 weeks after the last
intratracheal instillation of the carcinogen and then
were shifted to normal diet containing 20,000 IU
retinyl acetate kg-1 diet. In addition to BP-Fe203
treated rats, weight matched rats from normal and
vitamin A deficient groups were given 30 mg of
Fe203 intratracheally (10mg per instillation,
instillations one week apart) to serve as controls.
Rats were weighed and food consumption was
determined twice a week.

Animals were checked twice daily. Some rats
from each group died during study duration and
were found cannibalized or exhibited tissue
autolysis. These were not evaluated in the study.
Twenty or more rats from each group were
sacrificed at different time intervals (24, 28 and 32

weeks) after the last instillation of carcinogen. In
each case, lungs were removed along with the
trachea. Squamous nodules in lungs, >0.5cm or
<0.5cm were scored as end points in the study.
Lungs were fixed in 10% neutral buffered formalin,
processed for routine histological staining with
hematoxylin and eosin and examined micro-
scopically.

Liver vitamin A was estimated by the method of
Dugan et al. (1964). Statistical analysis of
differences was done by Student's t test.

Results

Table I presents the body weight and hepatic
vitamin A content of control and vitamin A
deficient groups at various time intervals in the
study. Initially, all groups were weight matched at
49 +6g per rat. Weight gain in group 3 was slightly
less than that in the other groups, but with the
overlap of standard deviation between group 3 and
other groups, this difference was not significant.
Hepatic vitamin A content in rats maintained on
vitamin A deficient diet for 4 weeks was reduced to
less than 2ugg -1 and was found to be 5pgg -1 in
the group of rats (Group 3) which was maintained
on a low vitamin A containing diet for a further 8
weeks after the final intratracheal instillation of BP.
However, hepatic vitamin A content in all the
groups sacrificed on 24, 28, and 32 weeks after the
last instillation of the carcinogen, did not show any
significant difference.

Table II shows tumour incidences in the different
groups, sacrificed at weeks 24, 28 and 32 after the

Table I Mean body weight and hepatic vitamin A content of rats maintained on diets with or without vitamin A

Average bodyeight (g) at                  Hepatic vitamin A content (pugg-1)' at

Group*    4 wk 6 wk 14 wk 30 wk 34 wk 38 wk         4 wk           14 wk      30 wk    34 wk    38 wk

1 (90)    148   170   251   300   315   320       90+12 (4)      74+13 (4)    71+14    66+11    69+13
2 (85)     142  162   242   285   303   302       1.8+0.5 (4)    66+11 (4)    70+12    65+9     64+7
3 (85)                233   292   305   302                     4.2+0.6 (4)   62+9     63+8     62+9
4 (70)     151  175   264   315   330   341       87+10 (4)      76?12 (4)    75+14    74+9     71+12
5 (70)    144   169   249   298   312   318       1.6+0.6 (4)   6.4+1.2 (4)   68+10    70+13    68+11

aMean+s.d. Number of rats used for estimating vitamin A content in liver at 4 and 14 weeks is given in parentheses.
Groups 1 through 3 received three intratracheal instillations, one week apart, of 10mg BP at 4 weeks after rats were
maintained on diets either containing 20,000 IU kg- 1 of retinyl acetate (group 1) or on vitamin A deficient diet (groups 2
and 3). At 6 weeks group 2 was shifted to diet containing 20,000IU of retinyl acetate kg- 1, whereas group 3 was fed diet
containing 1000IU of retinyl acetate kg-1 till 14 weeks and then this group was also shifted to the diet given to groups
1 and 2. Groups 4 and 5 were maintained on diets given to groups 1 and 3 respectively and received Fe203 instead of
BP+Fe203 so as to serve as controls. Weeks 30, 34 and 38 mentioned here corresponds to weeks 24, 28 and 32 after
the termination of BP+ Fe203 or Fe203 treatment.

*Values in parentheses are the number of animals in each group at first intratracheal instillation of BP+Fe203 or
Fe203.

VITAMIN A DEFICIENCY AND BP TUMOURIGENESIS

933

last BP-Fe2 03 intratracheal instillation. Tumours
were  verified  histopathologically  to  be  mainly

+- ''             squamous cell carcinomas (Figure 1). LDetall

typing of tumours was not carried out. The
011,0   0     ,majority   of the lung   tumours were roughly
o Q, E  3        spherical and symmetrical (Figure 2). The mean

0 0   C        tumour diameter and number of tumours per
<,, t4n  N    tumour-bearing animal (TBA) indicate the relative

degrees of tumour burden in the various groups.
Fe203 treated groups (Groups 4 and 5) in which
=  . <,  F   rats were maintained on the same diets as the rats
-o Y a  Oin groups 1 and 3 respectively, did not reveal any
< ,,~ X    ,,,   tumours even after 32 weeks. At 24 weeks, rats

from group 1 had the lowest tumour incidence and
| 3  o 0     T/TBA ratio, whereas these parameters were higher

> >            0< in groups 2 and 3, showing maximum incidence in
-o   U        groun 3 which was kept on low vitamin A diet after

o ot    +         the last BP-Fe203 dose. At 28 weeks, tumour

-  r  ;          incidence increased in all three groups but it still

q     4. Oremained far below that in group 1 as compared to
enz>rQ  C<;:  groups 2 and 3. Rats in group 3 showed 100%
.0= <,,"  ?tumour incidence, however the T/TBA ratio in all
= E +    <         the groups remained almost the same as it was at
a,                24 weeks. At 32 weeks, group 1 did not show any
CO ;O    ,further increase in tumour incidence, whereas it

increased from 75%  to 83%  in group 2, and was
100% in group 3. At this point the T/TBA ratio
<,, g =  e        was increased in all groups, thereby revealing an

increased tumour burden per rat. The mortality due

to intratracheal instillations of BP-Fe2 03 was found

1.0~~~~~~~~~~

&O       C)
? oC0    .

00     0

cO

00

0

Figure 1 Well differentiated squamous cell carcinoma
with horny pearls. H & E ( x 33).

* )

.E

.,

CO

-o

0
._
0

00

0

0

0
0
CO

0

.0

C)

a)

4 W

a    E
z-   E

A

Q    S
0    V

.p i

a)    "R  -
0-    c

-0        E

0        E

L.

a)   4

3 A

0_    m

bt     V

z 4
-0 c

0      E

o  oo I  I

'IO 00 00 II
<00 I I

en   I  I

N en 8 00

en 000
I   s 0

2oo     I I
0 00    I

cq  4   I   I

I. I 1- _ -   I-   I.-
(N (N N N (N

t     0Q  0 o   Q
eq . .o

_-1I-   --  -   -

en2en   W)  I  I

0  0 C o   I  I

't   t I  I

0    0   ' ( I   I

CQ 0 n (N (N

-q (N C1 e C4-

I-   _.-   I-   -   -
c, s 0ooc
en   0 Ooo

1o    '  -C4c

CO

*-j

C)

-o
0

0

00
0
0

*

0,

r-

03

-?e
Rt
N
erl

I

I .

-,Ad

A4

I

I                                                     I

I

-4d
k

934     S.C. DOGRA et al.

Figure 2 Gross appearance of a large squamous cell
tumour in the middle portion of the right lung and
with multiple tumours in the left lung. Rats received
three intratracheal instillations, one week apart, of

1Omg BP. Experimental period 28 weeks.

to be maximal in animals kept on vitamin A
deficient diet.

Discussion

The role of vitamin A deficiency in tumourigenesis
has   been   studied  by   several  investigators
(Nettesheim & Williams, 1976; Rogers et al., 1973;
Sporn & Newton, 1981). However, the exact
mechanism by which vitamin A influences this
process has not been well delineated. In our present
study, on periodically killing of rats treated with
BP, we have observed that the tumour incidence in
vitamin A deficient animals was more than double
that in normal pairfed rats. This enhanced
susceptibility of the lung to BP-induced carcino-
genesis in vitamin A deficiency could possibly be
related to a number of factors. Firstly, as changes
inflicted by vitamin A deficiency have been shown
to be similar to precancerous lesions, vitamin A
deficiency may therefore, elevate the initiation and
progression of tumourigenic process in animals
exposed to chemical carcinogens, as has been
observed in our study. Secondly, predisposition of
the lung to chemical carcinogenesis may be due to
an altered pattern of enzymes involved in the

metabolism of these carcinogens, both in lung and
liver. BP, like many other polycyclic aromatic
hydrocarbons, requires metabolic activation by the
action of mixed-function oxidases and vitamin A
status has been shown to alter the activities of these
enzymes (Becking, 1973; Miranda et al., 1979;
Siddik et al., 1980). Our earlier studies in this area
(Dogra et al., 1982; Dogra et al., 1983) suggested
that imbalance between activation and conjugation
processes in lung and liver in vitamin A deficiency
might result in an increased yield of carcinogenic
metabolites and their slow elimination from the
body. This might, in turn, produce a favourable
environment for the enhanced binding of reactive
carcinogenic metabolites with critical cellular
macromolecules like DNA. Earlier, we observed
enhanced binding of [3H]-BP to lung DNA    in
vitamin A deficient rats in in vivo and in vitro
studies (Dogra et al., 1984). These studies give
support to the argument that mixed-function
oxidase enzymes play a very crucial and important
role in determining organ susceptibility to
tumourigenesis.

In the present study vitamin A deficiency has
been  found   to   enhance  the   process  of
tumourigenesis, both at the initiation and post-
initiation phases. However, the effect of vitamin A
deficiency was more prominent at the initiation
phase as rats from group 2, killed at 32 weeks after
the termination of BP instillation had 83% tumour
incidence compared to 39% in normal pairfed rats
(group 1). Moreover, the tumour burden per rat
was also greater in vitamin A deficient rats than in
normal pairfed rats. Furthermore, the enhancing
effect of vitamin A deficiency on carcinogenesis
during the postinitiation phase was evident from
the data on group 3 as rats which were kept on
vitamin A deficient diet during and after the
challenge with BP, not only had a higher
percentage tumour incidence but also the tumour
burden per rat was even greater. Also, this higher
incidence of tumours was achieved earlier than in
the other two groups (groups 1 and 2). This could
be due to lack of vitamin A as an essential factor
for the normal differentiation of cells during the
post initiation phase of tumourigenesis. The other
responsible factor could be the slow elimination of
carcinogen (given as BP-Fe203) from  the lung.
Therefore, an effect of vitamin A deficiency on the
metabolism of the carcinogen and the penetration
of the carcinogens into the target cells could not be
ruled out.

In conclusion this study has revealed that vitamin
A deficiency enhances the process of lung
carcinogenesis induced by benzo(a)pyrene both at
the initiation and postinitiation phases. However,
the more pronounced effect was seen at the
initiation stage.

VITAMIN A DEFICIENCY AND BP TUMOURIGENESIS  935

References

BECKING, G.C. (1973). Vitamin A status and hepatic

drug-metabolism in the rat. Can. J. Physiol.
Pharmacol., 51, 6.

BOLLAG, W. & MATrER, A. (1981). From vitamin A to

retinoids in experimental and clinical oncology. Ann.
N.Y. Acad. Sci., 27, 9.

DOGRA, S.C., KHANDUJA, K.L. & SHARMA, R.R. (1982).

Effect of vitamin A deficiency on the level of
glutathione and glutathione S-transferase activity in
rat lung and liver. Experientia, 38, 903.

DOGRA, S.C., KHANDUJA, K.L., GUPTA, M.P. & SHARMA,

R.R. (1983). Effect of vitamin A deficiency on the
pulmonary and hepatic drug-metabolizing enzymes in
rat. Enzyme, 30, 99.

DOGRA, S.C., KHANDUJA, K.L., GUPTA, M.P. & SHARMA,

R.R. (1984). Binding of benzo(a)pyrene to DNA in
normal and vitamin A deficient rats. Ind. J. Biochem.
Biophysics, 21, 117.

DUGAN, R.E., FRIGERIO, N.A. & SIEBERT, J.M. (1964).

Colourimetric determination of vitamin A and its
derivatives with trifluoroacetic acid. Anal. Chem., 36,
114.

HARRIS, C.C., SPORN, M.B., KAUFMAN, D.G., SMITH,

J.M., JACKSON, F.E. & SAFFIOTTI, U. (1972). Histo-
genesis of squamous metaplasia in the hamster trachea
epithelium caused by vitamin A deficiency or
benzo(a)pyrene-Ferric oxide. J. Natl Cancer Inst., 48,
743.

HAYES, K.C. (1971). On the pathogenesis of vitamin A

deficiency. Nutr. Rev., 29, 3.

LOTAN, R. (1980). Effects of vitamin A and its analogs on

normal and neoplastic cells. Biochem. Biophys. Acta,
605, 33.

MIRANDA, C.L., MUKHTAR, H., BEND, J.R., CHHABRA,

R.S. (1979). Effects of vitamin A deficiency on hepatic
and extrahepatic mixed-function oxidase and epoxide-
metabolizing enzymes in guinea pig and rabbit.
Biochem. Pharmacol., 28, 2713.

NETTESHEIM, P., CONE, V. & SYNDER, C. (1976). The

influence of retinyl acetate on the postinitiation phase
of lung cancer in rats. Cancer Res., 36, 996.

NETTESHEIM, P. & WILLIAMS, M.L. (1976). The influence

of vitamin A on the susceptibility of the rat lung to 3-
methylcholanthrene. Int. J. Cancer, 17, 351.

ROGERS, A.E., HERNDON, B.J. & NEWBERNE, P.M.

(1973). Induction of intestinal carcinoma in normal
rats and rats fed high or low levels of vitamin A.
Cancer Res., 33, 1003.

SIDDIK, Z.H., DREW, R., LITTERST, C.L., MIMNAUGH,

E.G., SIKIC, B.I. & GRAM, T.E. (1980). Hepatic
cytochrome   P-450  dependent   metabolism   and
enzymatic conjugation of foreign compounds in
vitamin A deficient rats. Pharmacology, 21, 383.

SPORN, M.B. (1976). Approaches to prevention of

epithelial cancer during the preneoplastic period.
Cancer Res., 36, 2699.

SPORN, M.B. & NEWTON, D.L. (1981). Retinoids and

chemoprevention of cancer. In Inhibition of tumor
induction and development, Zedeck, M.S. and Lipkin,
M. (eds) p. 71. Plenum Publishing Corp.: New York.

WOLBACK, S.B. (1954). Effects of vitamin A deficiency

and hypervitaminosis A in animals. In The vitamins,
Sebrell, W.H. & Harris, R.S. (eds) p. 106, vol. I.
Academic Press: New York.

				


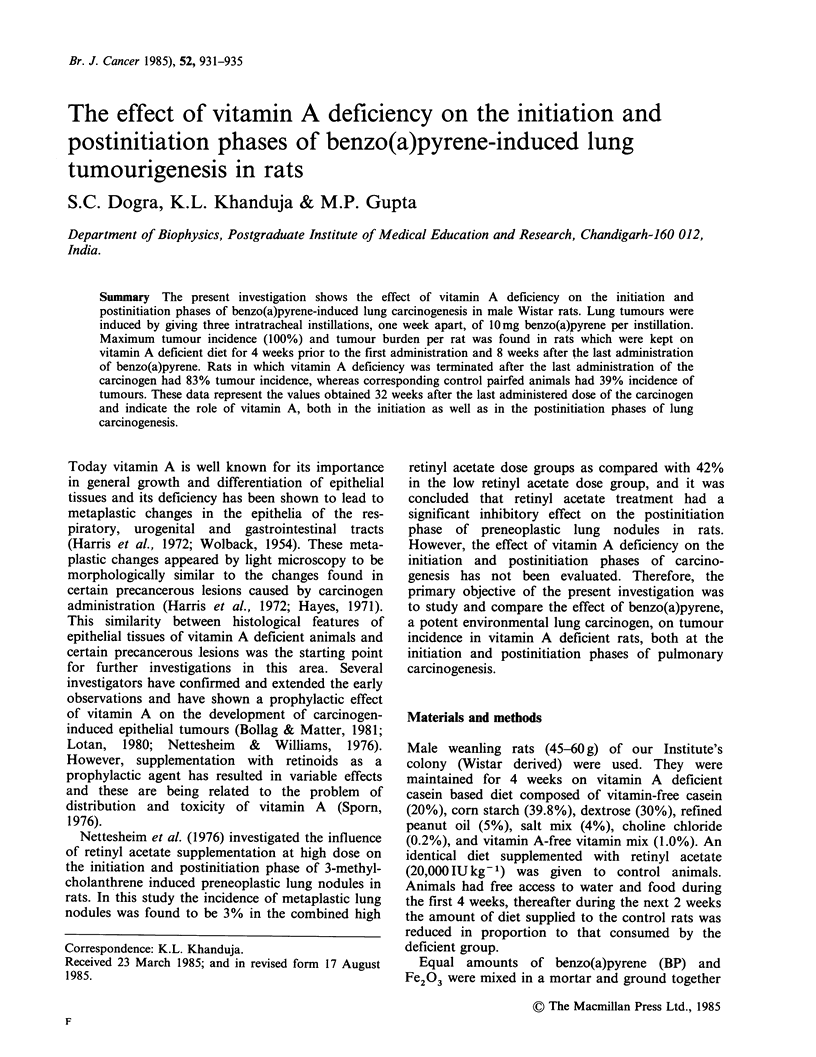

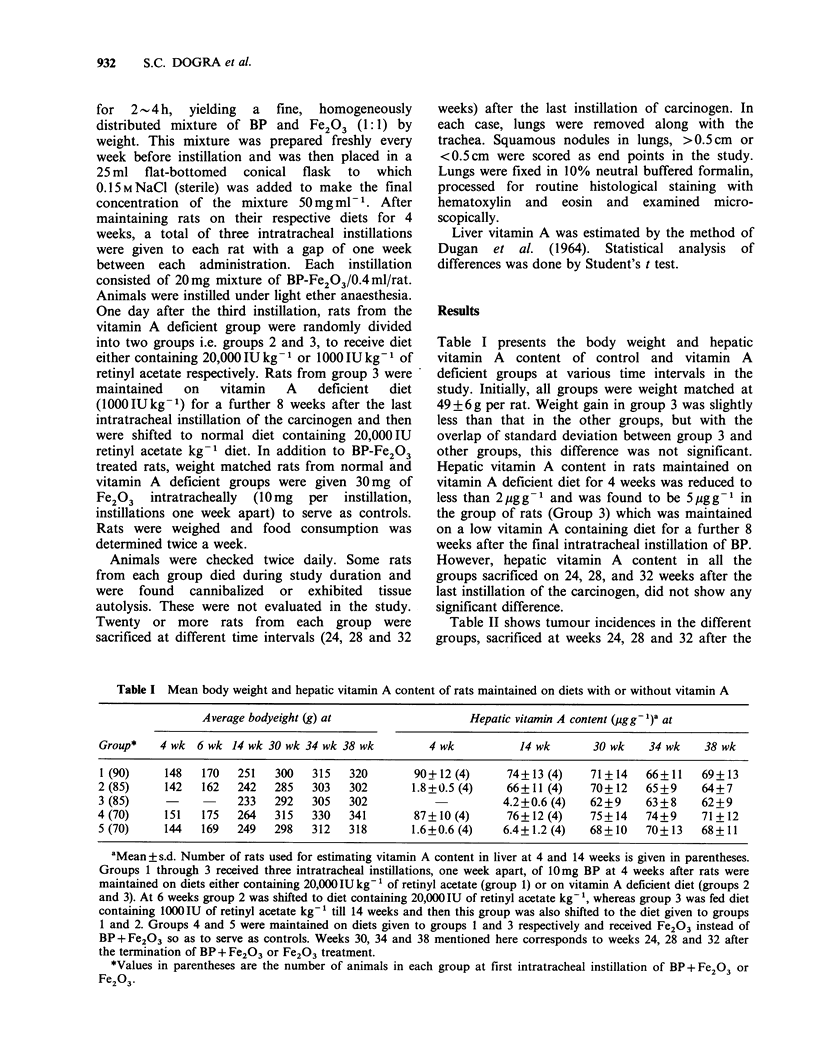

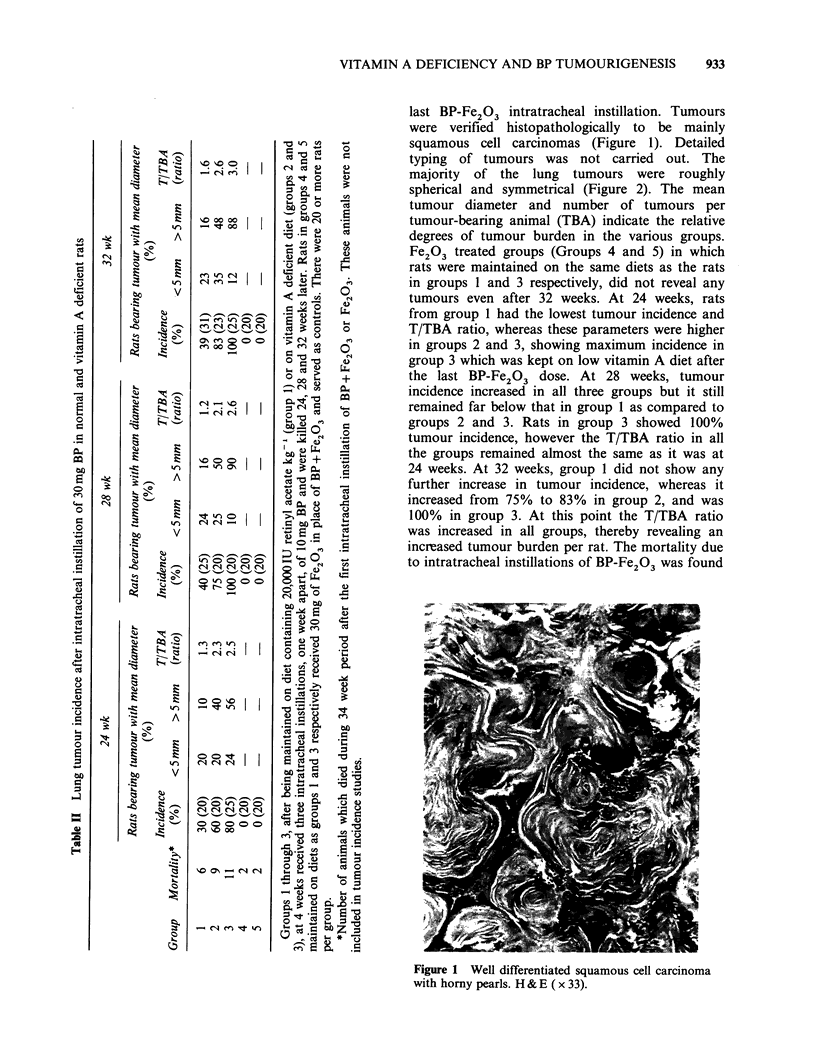

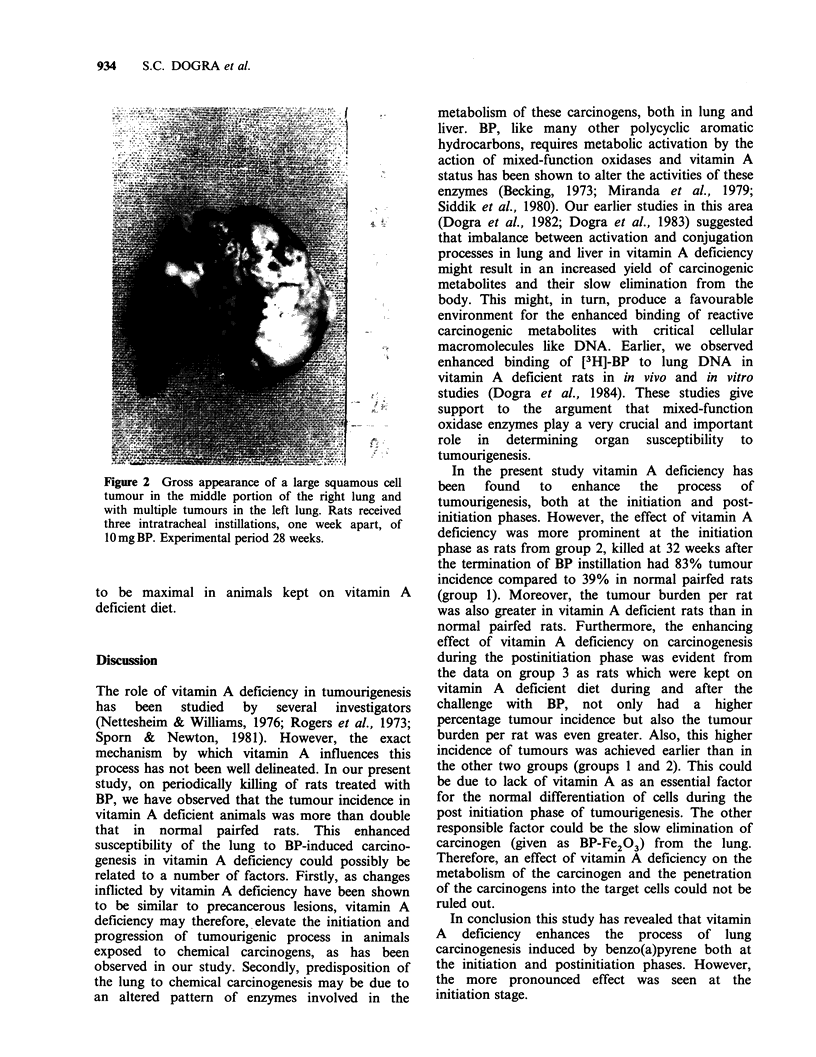

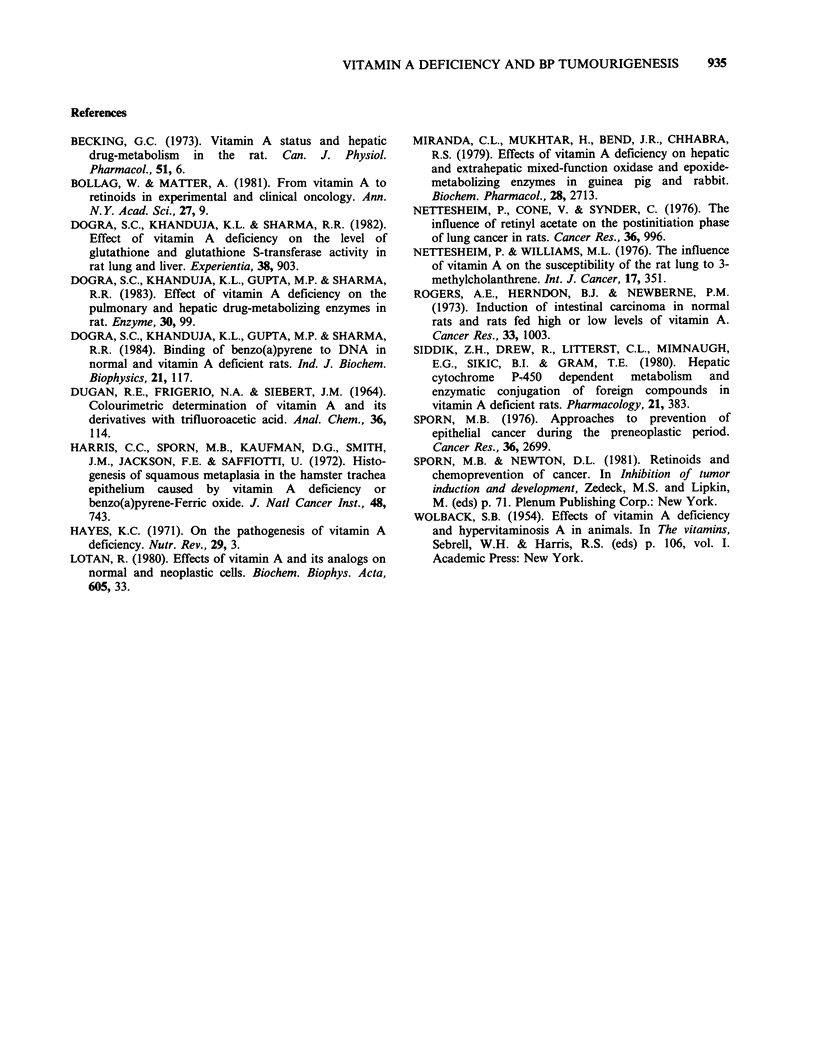

